# Development of a rule-based automatic five-sleep-stage scoring method for rats

**DOI:** 10.1186/s12938-019-0712-8

**Published:** 2019-09-04

**Authors:** Ting-Ying Wei, Chung-Ping Young, Yu-Ting Liu, Jia-Hao Xu, Sheng-Fu Liang, Fu-Zen Shaw, Chin-En Kuo

**Affiliations:** 1 Dept. of Computer Science and Information Engineering, National Cheng Kung University, Tainan, 701 Taiwan; 2 Department of Medical Sciences Industry, Chang Jung Christian University, Tainan, 711 Taiwan; 3 Department of Psychology, National Cheng Kung University, Tainan, 701 Taiwan; 4 Department of Automatic Control Engineering, Feng Chia University, Taichung, 407 Taiwan

**Keywords:** Rat, Automated sleep scoring, Five sleep stages, Rule-based, Decision tree

## Abstract

**Background:**

Sleep problem or disturbance often exists in pain or neurological/psychiatric diseases. However, sleep scoring is a time-consuming tedious labor. Very few studies discuss the 5-stage (wake/NREM1/NREM2/transition sleep/REM) automatic fine analysis of wake–sleep stages in rodent models. The present study aimed to develop and validate an automatic rule-based classification of 5-stage wake–sleep pattern in acid-induced widespread hyperalgesia model of the rat.

**Results:**

The overall agreement between two experts’ consensus and automatic scoring in the 5-stage and 3-stage analyses were 92.32% (*κ* = 0.88) and 94.97% (*κ* = 0.91), respectively. Standard deviation of the accuracy among all rats was only 2.93%. Both frontal–occipital EEG and parietal EEG data showed comparable accuracies. The results demonstrated the performance of the proposed method with high accuracy and reliability. Subtle changes exhibited in the 5-stage wake–sleep analysis but not in the 3-stage analysis during hyperalgesia development of the acid-induced pain model. Compared with existing methods, our method can automatically classify vigilance states into 5-stage or 3-stage wake–sleep pattern with a promising high agreement with sleep experts.

**Conclusions:**

In this study, we have performed and validated a reliable automated sleep scoring system in rats. The classification algorithm is less computation power, a high robustness, and consistency of results. The algorithm can be implanted into a versatile wireless portable monitoring system for real-time analysis in the future.

## Background

Mammals display different patterns of sleep–wake stages, which comprise cyclic patterns of wakefulness (wake), non-rapid eye movement (NREM) sleep and rapid eye movement (REM) sleep. These stages have been defined based on electrophysiological measurements that include electroencephalogram (EEG), electromyogram (EMG), and electrooculogram (EOG). Many diseases and cognitive behaviors have a relationship with sleep quality and quantity. For instance, patients with chronic widespread pain syndromes often exhibit sleep disturbance [1]. These patients usually complain unrefreshing sleep, i.e., too much light sleep or irregular sleep pattern [2]. To understand the pathogenesis of pain or comorbidity of sleep disturbance, an ideal animal model may be required. In the field of sleep research, various rat models are often used because they are readily available and display electrical activity during sleep that has similarities with human sleep [3–8].

Clinically, a patient claims a long suffering muscle pain but found no damage in body tissue after checking, this patient will be diagnosed as fibromyalgia syndrome according to American College of Rheumatology diagnostic criteria: if the pain site throughout the body, especially in the vicinity of the joint hyperalgesia phenomena. Fibromyalgia and sleep disorders also go hand in hand. In fact, it is thought that up to 80% of patients with fibromyalgia experience certain type of disordered sleep. Often, these sleep disorders leave people feeling tired, drained, and physically incapable of dealing with the stresses associated with fibromyalgia [9]. Sluka et al. [10] have proposed an animal model of widespread mechanical hyperalgesia lasting for about 4 weeks by repeated intramuscular injections of pH 4.0 saline. However, sleep recording is not assessed in this model.

One of the major inconveniences encountered in sleep studies is the time-consuming labor involved in equating the visual analysis of physiological recordings (EEG, EMG, and EOG) to an appropriate stage of vigilance. Most previous automatic sleep staging methods for rats have distinguished three main stages (i.e., wake, NREM and REM) [11–17]. However, to obtain a more detailed analysis in different sleep stages, numerous previous studies have proposed to utilize a four or five-stage analysis for wake–sleep pattern in rats [18–21]. Therefore, developing an automated system that can distinguish five wake–sleep stages will be beneficial to investigate subtle change of wake–sleep pattern in response to different behavioral changes.

**Fig. 1:**
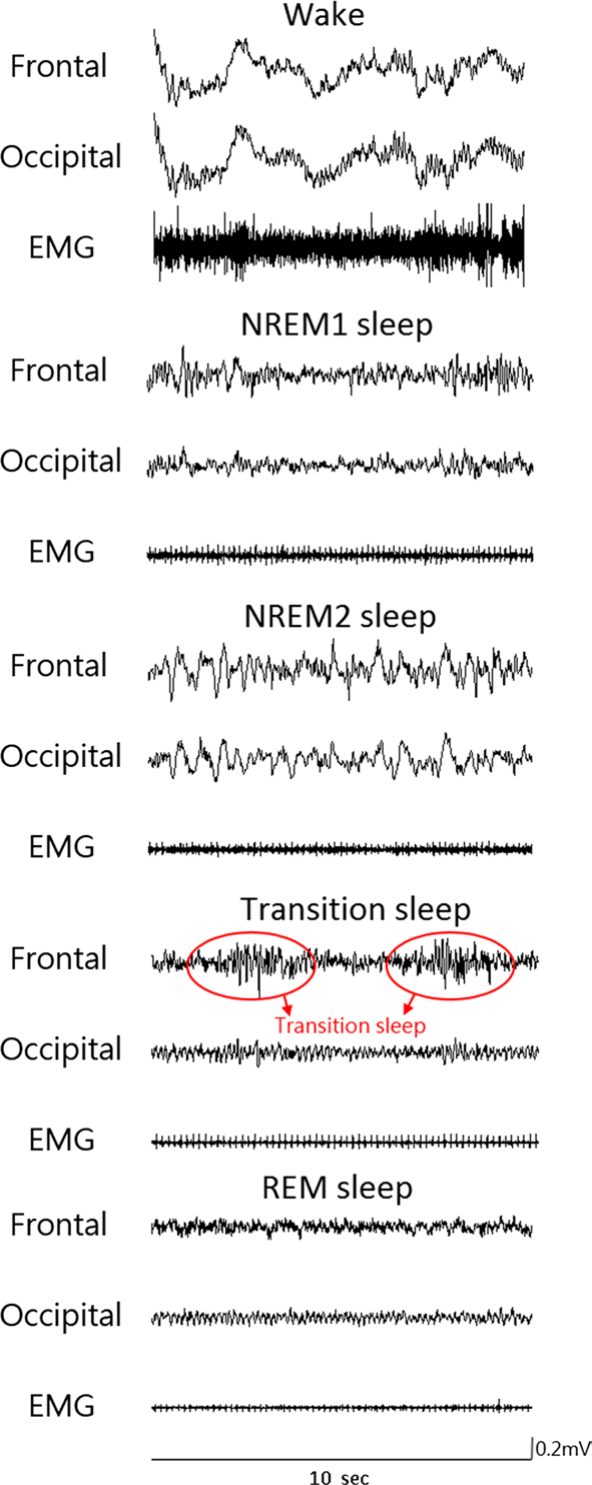
Typical polygraphic recordings during the different five sleep stages. Frontal, electroencephalogram (EEG) of frontal; Occipital, electroencephalogram (EEG) of occipital; NREM, non-rapid eye movement; REM, rapid eye movement

Since the standard sleep staging rules for rats are not available currently, we referred the visual scoring criteria from two previous studies [20, 22]. Five stages, including wake, NREM sleep stage 1 (NREM1), NREM sleep stage 2 (NREM2), transition sleep (TS), and REM sleep, were adopted in this study. Figure 1 shows typical polygraphic recordings representing the five stages. In the wake stage, EEG exhibits low-amplitude predominant theta activity (6–9 Hz) and superimposed high-frequency activity accompanied by a large amplitude EMG. In the NREM1 stage, sleep spindles (10.5–15 Hz) and/or median delta wave activity (0.5–5 Hz) less than 50% of an epoch are present, and EMG exhibits a diminished amplitude compared with the wake stage. In the NREM2 stage, high delta wave (0.5–5 Hz) activity occupied more than 50% of an EEG epoch, and EMG amplitude diminishes compared with the NREM1 sleep. In the TS stage, EEG of parietal or occipital lead exhibits a prominent theta rhythm intermixed with short-lasting (1–3 s) high-amplitude spindles, and EMG amplitude was low [16]. In the REM sleep, EEG exhibits a regular theta rhythm, and EMG is absent (often presentation of electrocardiography) or with the exception of occasional short-lasting bursts of activity typically associated with rapid eye movements.

The classification procedures rely on highly refined methods, such as cluster analysis [23], linear discriminant analysis [19], artificial neural networks [24], pattern matching algorithm [25, 26] and support vector machine (SVM) [14]. Previous studies [24, 27] indicate that artificial intelligence is a best approach to accurately score sleep stages allowing to adapt to the various scoring requirements of different researchers. Thus, a customizable rule-based auto-scoring program is well suited for a researcher who would prefer more control over the decision-making process for scoring stages rather than exclusive reliance on the program. Here, we proposed a rule-based automatic five-sleep-stage scoring method that was constructed using a hierarchical decision tree. According to characteristics of biosignals and staging rules, several modifications were used. The present study introduced a two-stage process in the hierarchical decision tree to increase staging accuracy. Ten features, including temporal and spectrum analyses of the EEG and EMG signals, were utilized [16, 27]. Normalization of the EEG index was applied to eliminate individual differences and make the distribution of the features to be centralized. Because the EMG signal only indicated the movement situation and only used as an references for the discrimination of the wake stage. The classification accuracy was tested with a large dataset (20 sets of 24-h recordings) comparing with visual scoring from two experts. In addition, the performances, including overall agreement and kappa coefficient of five and three stages, were compared to the existing methods. The present study further aimed to validate effectiveness of the 5-stage or 3-stage analyses on sleep disturbance of the acid-induced hyperalgesia model.

## Results

### The performance of 5-stage scoring

The confusion matrix of the 5-stage scoring between the expert consensus and automatic scoring from 168,656 epochs of 20 rats is shown in Table 1. The sensitivities of the wake, NREM1, NREM2, TS, and REM stages were 94.4%, 91.27%, 91.26%, 78.98%, and 90.48%, respectively. The specificities of the wake, NREM1, NREM2, TS, and REM stages were 99.96%, 94.57%, 98.8%, and 99.2%, respectively. Almost all indexes between the automatic scoring method and expert consensus attained 90%. The overall agreement was 92.32%. The kappa coefficient was 0.88, which indicated an excellent agreement.

**Table 1:** Confusion matrix of the 5-stage analysis between the automatic staging method and expert consensus

	Automatic staging					
	Wake	NREM1	NREM2	TS	REM	Total
Expert consensus						
Wake	77,822	1747	231	154	486	80,440
NREM1	3807	41,452	1576	718	591	48,144
NREM2	499	1207	19,663	5	60	21,434
TS	152	907	51	4013	206	5329
REM	156	175	24	191	12,763	13,309
Total	82,436	45,488	21,545	5081	14,106	168,656
SE (%)	94.4	91.13	91.26	78.98	90.48	
SP (%)	96.96	94.57	98.8	99.2	99.65	
PPV (%)	96.75	86.1	91.74	75.3	95.9	
NPV (%)	94.77	96.65	98.72	99.35	99.14	
Overall agr. (%)	92.32					
Kappa	0.88					

### The performance of 3-stage scoring

The confusion matrix of the 3-stage scoring between the expert consensus and automatic scoring from 168,656 epochs of 20 rats is reported in Table 2. The sensitivities of wake, NREM, and REM were 94.4%, 96.5%, and 90.48%, respectively. The specificities of wake, NREM, and REM were 96.96%, 94.49%, and 99.64%, respectively. All indexes between the automatic scoring method and expert consensus were greater than 90%. The overall agreement was 94.97%. The kappa coefficient was 0.91, which indicated an excellent agreement.

**Table 2:** Confusion matrix of the 3-stage analysis between the automatic staging method and expert consensus

	Automatic staging			
	Wake	NREM	REM	Total
Expert consensus				
Wake	77,822	2132	486	80,440
NREM	4458	69,592	857	74,907
REM	156	395	12,763	13,309
Total	82,436	72,114	14,106	168,656
SE (%)	94.4	96.5	90.48	
SP (%)	96.96	94.49	99.64	
PPV (%)	96.75	92.9	95.86	
NPV (%)	94.77	97.3	99.14	
Overall agr. (%)	94.97			
Kappa	0.91			

### Individual performance

Table 3 shows the agreements and kappa coefficients between the expert consensus scoring and automatic scoring in all subjects using the 5-stage and 3-stage analyses. We firstly considered performance of the 5-stage analysis. Agreement fell in the range of 87.42–97.18%. Fourteen subjects (70% of 20 rats) exhibited agreement of > 90%. Averaged agreement was 91.94%. The kappa coefficient was in the range of 0.78–0.96. Nineteen subjects (95% of 20 rats) exhibited an excellent agreement (i.e., *κ* > 0.80). Averaged kappa coefficient was 0.87 for the 5-stage analysis.

**Table 3:** Agreement and Cohen’s kappa coefficient between the expert consensus and automatic scoring in all individuals under the 3-stage and 5-stage analyses

Subject	Agr. of 5-stage (%)	*κ* of 5-stage	Agr. of 3-stage (%)	*κ* of 3-stage
No. 1	92.17	0.87	93.64	0.88
No. 2	91.79	0.88	94.5	0.91
No. 3	91.07	0.87	95	0.91
No. 4	89.92	0.83	92.3	0.86
No. 5	91.67	0.87	95.09	0.91
No. 6	93.61	0.90	96.6	0.94
No. 7	94	0.91	96.66	0.94
No. 8	90.16	0.85	92.75	0.87
No. 9	92.08	0.88	94.38	0.90
No. 10	90.31	0.85	91.65	0.85
No. 11	97.18	0.96	98.64	0.96
No. 12	96.84	0.95	98.87	0.98
No. 13	96.97	0.95	97.9	0.96
No. 14	94.85	0.92	95.73	0.92
No. 15	89.89	0.84	92.31	0.86
No. 16	87.42	0.81	90.22	0.82
No. 17	88.6	0.84	92	0.86
No. 18	87.95	0.82	91.77	0.85
No. 19	93.09	0.9	97.33	0.95
No. 20	89.32	0.78	90.44	0.8
Mean	91.94	0.87	94.39	0.90

In the 3-stage scoring, agreement between the expert consensus scoring and automatic scoring fell in the range of 90.22–98.87%. All subjects (100%) exhibited agreement of > 9 = 0%. Averaged agreement was 94.39%. The kappa coefficient was in the range of 0.8–0.98. All subjects (100%) exhibited an excellent agreement. Averaged kappa coefficient was 0.90. These results demonstrated that the proposed rule-based method in either the 5-stage or 3-stage analysis achieved stable high performance.

### Sleep disturbance on acid-induced hyperalgesic model

**Fig. 2:**
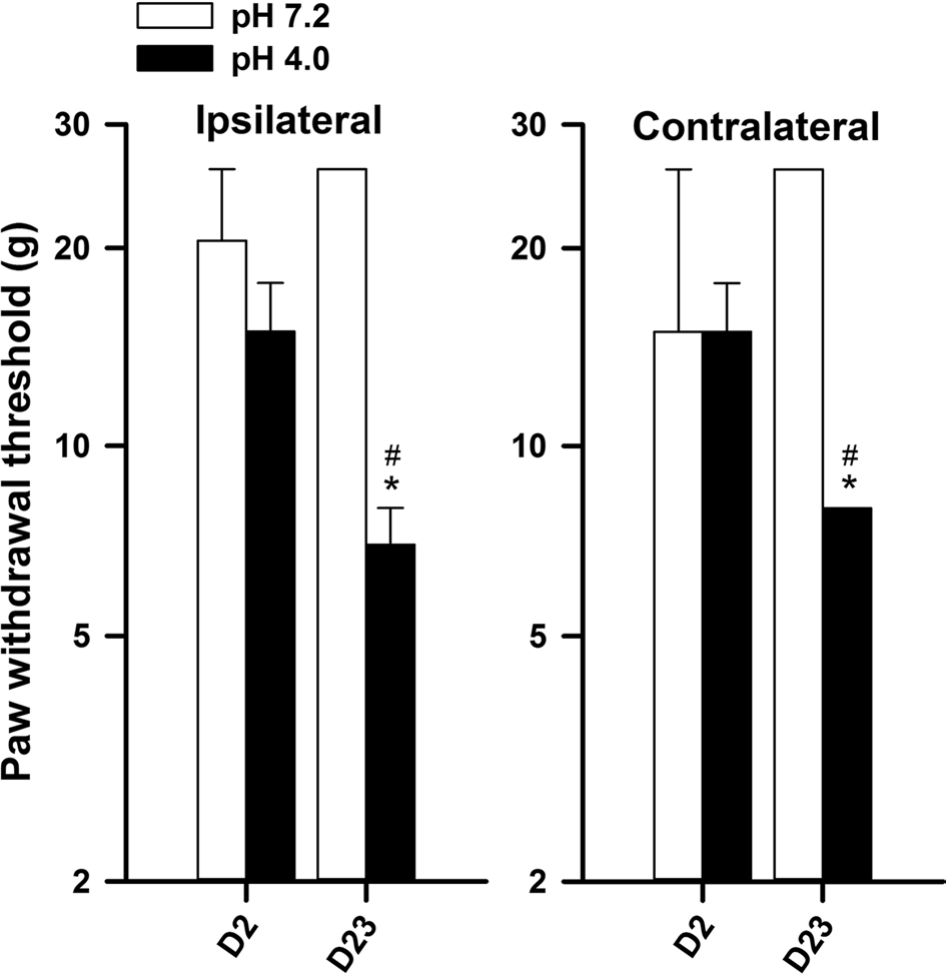
Changes of paw withdrawal thresholds in bilateral hindlimbs in the groups receiving pH 7.2 saline or pH 4.0 saline at day 2 (D2, baseline) and day 23 (2 weeks after the 2nd injection). *p < 0.05 compared with D2; ^#^*p *< 0.05 compared with the group of pH 7.2

Figure 2 shows paw withdrawal thresholds of bilateral hindlimbs at days 2 and 23 in both vehicle group and acid group. Paw withdrawal thresholds of ipsilateral hindlimb exhibited significant difference in the factors of treatment (*F*_1,18_ = 22.355, *p *< 0.001), time (*F*_1,18_ = 11.631, *p *= 0.003), and time × treatment (*F*_1,18_ = 14.979, *p *= 0.001). Paw withdrawal threshold of the acid group at day 23 revealed significantly lower than that of the vehicle group or that before the injection of pH 4.0 saline. Paw withdrawal thresholds of contralateral hindlimb exhibited significant difference in the factors of treatment (*F*_1,18_ = 23.869, *p *< 0.001), time (*F*_1,18_ = 5.339, *p *= 0.033), and time × treatment (*F*_1,18_ = 11.678, *p *= 0.003). Paw withdrawal threshold of the acid group at day 23 revealed significantly lower than that of the vehicle group or that before the injection of pH 4.0 saline. The results indicated that repetitive injections of pH 4.0 saline into unilateral hindlimb caused bilateral hindlimb hyperalgesia.

**Fig. 3:**
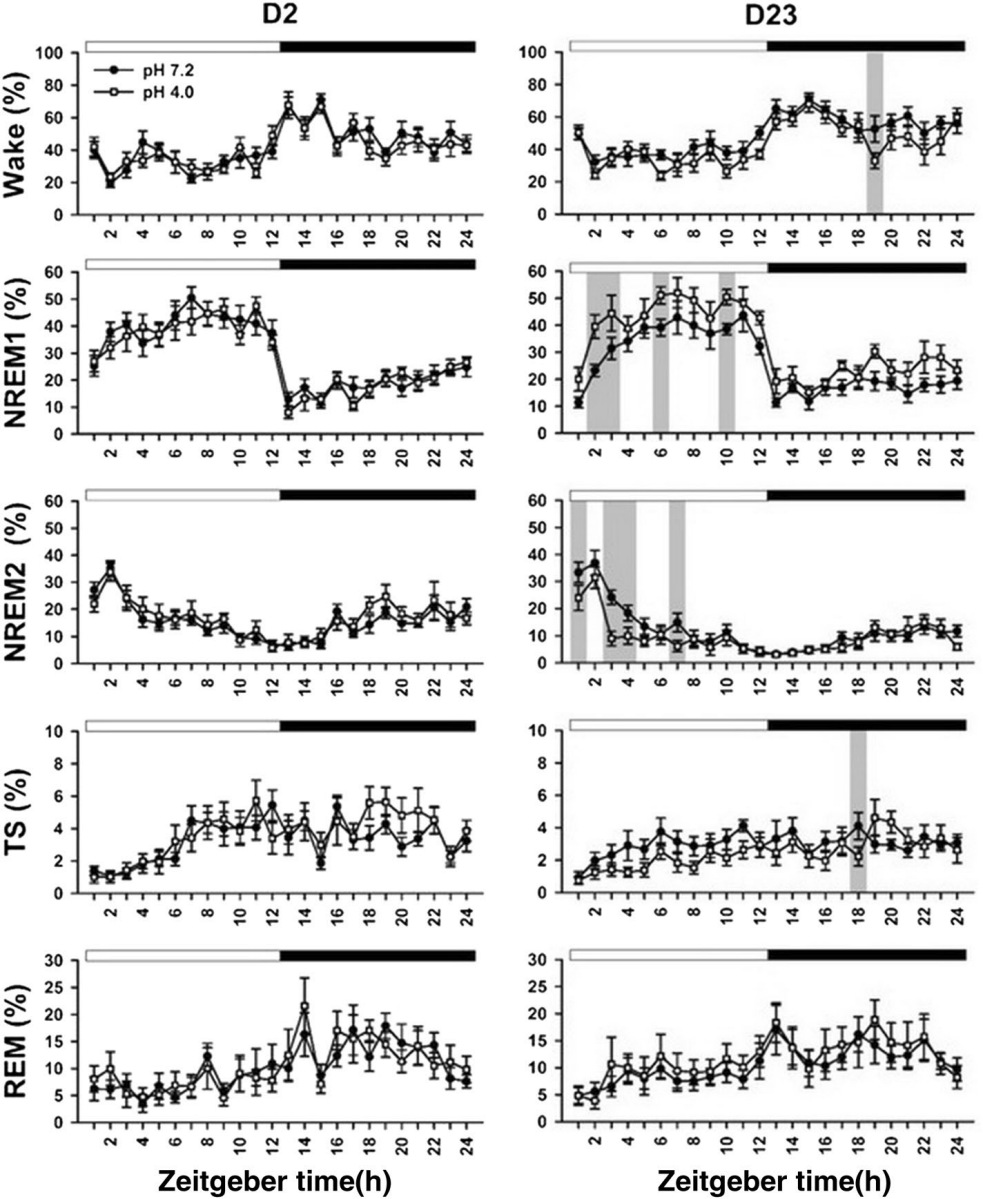
Changes of 5 wake–sleep stages per hour in the light and dark periods at days 2 and 23. Statistical significance between the vehicle group (filled circle) and acid group (open square) is indicated by gray bar. Light and dark periods are indicated with open horizontal bar and filled horizontal bar at the top of each subplot, respectively. Time is modified as zeitgeber time

Figure 3 shows 5-stage wake–sleep changes of the two groups at day 2 and day 23. High portion of the wake stage occurred at dark period, and sleep stage often exhibited at light period. In particular, NREM2 sleep primarily occurred at the early phase of the light period followed by abundant NREM1 sleep at the late phase of the light period. Table 4 summarizes all statistical results of 5-stage wake–sleep changes between the two groups at 2 timepoints. There was significant difference in the factor of time exclusively at day 2. In a sharp contrast to day 2, there was significant difference in the factors of time in all parameters, treatment in wake and NREM1, and time × treatment in NREM2 at day 23. At day 23, the two groups exhibited significant difference in the wake and TS at a particular timepoint of the dark period. The acid group exhibited longer NREM1 and shorter NREM2 in the light period compared with those of the vehicle group. Table 5 shows durations of the NREM1 and NREM2 in the light period of days 2 and 23 in the two groups. There was no significant difference in durations of the NREM1 and NREM2 between the two groups at day 2. In contrast, NREM1 duration of the acid group was significantly longer than that of the vehicle group at day 23. NREM2 duration of the acid group was significantly shorter than that of the vehicle group.

**Table 4:** Summary of statistical results of wake–sleep stages throughout the recording

Stage	Factor		
	Treatment	Time	Time × treatment
D2			
Wake	*F* = 0.20 (*p *= 0.657)	*F* = 10.28 (*p *< 0.001)*	*F* = 0.68 (*p *= 0.864)
NREM1	*F* = 0.13 (*p *= 0.720)	*F* = 24.23 (*p *< 0.001)*	*F* = 0.73 (*p *= 0.816)
NREM2	*F* = 0.26 (*p *= 0.618)	*F* = 12.67 (*p *< 0.001)*	*F* = 0.70 (*p *= 0.844)
TS	*F* = 0.49 (*p *= 0.492)	*F* = 5.80 (*p *< 0.001)*	*F* = 0.92 (*p *= 0.574)
REM	*F* = 0.02 (*p *= 0.889)	*F* = 5.78 (*p *< 0.001)*	*F* = 0.70 (*p *= 0.846)
NREM (NREM1 + NREM2 + TS)	*F* = 0.27 (*p *= 0.612)	*F* = 18.25 (*p *< 0.001)*	*F* = 0.91 (*p *= 0.584)
D23			
Wake	*F* = 4.95 (*p *= 0.039)*	*F* = 10.14 (*p *< 0.001)*	*F* = 0.78 (*p *= 0.764)
NREM1	*F* = 6.51 *(p *= 0.020)*	*F* = 22.51 (*p *< 0.001)*	*F* = 0.64 (*p *= 0.903)
NREM2	*F* = 2.26 (*p *= 0.150)	*F* = 19.41 (*p *< 0.001)*	*F* = 1.82 (*p *= 0.012)*
TS	*F* = 2.82 (*p *= 0.111)	*F* = 2.52 (*p *< 0.001)*	*F* = 1.10 (*p *= 0.339)
REM	*F* = 0.20 (*p *= 0.658)	*F* = 4.19 (*p *< 0.001)*	*F* = 0.27 (*p *= 1.00)
NREM (NREM1 + NREM2 + TS)	*F* = 0.27 (*p *= 0.612)	*F* = 18.25 (*p *< 0.001)*	*F* = 0.91 (*p *= 0.584)

* *p *< 0.05

**Table 5:** Durations of NREM1 and NREM2 sleep in the light period

Stage	Factor			
	Day 2		Day 23	
	Vehicle	Acid	Vehicle	Acid
NREM1 (h)	7.01 ± 0.46	6.80 ± 0.33	6.15 ± 0.50	7.97 ± 0.51*
NREM2 (h)	3.73 ± 0.30	3.87 ± 0.50	2.90 ± 0.26	2.32 ± 0.29*

* *p *< 0.05, vs. vehicle group

**Fig. 4:**
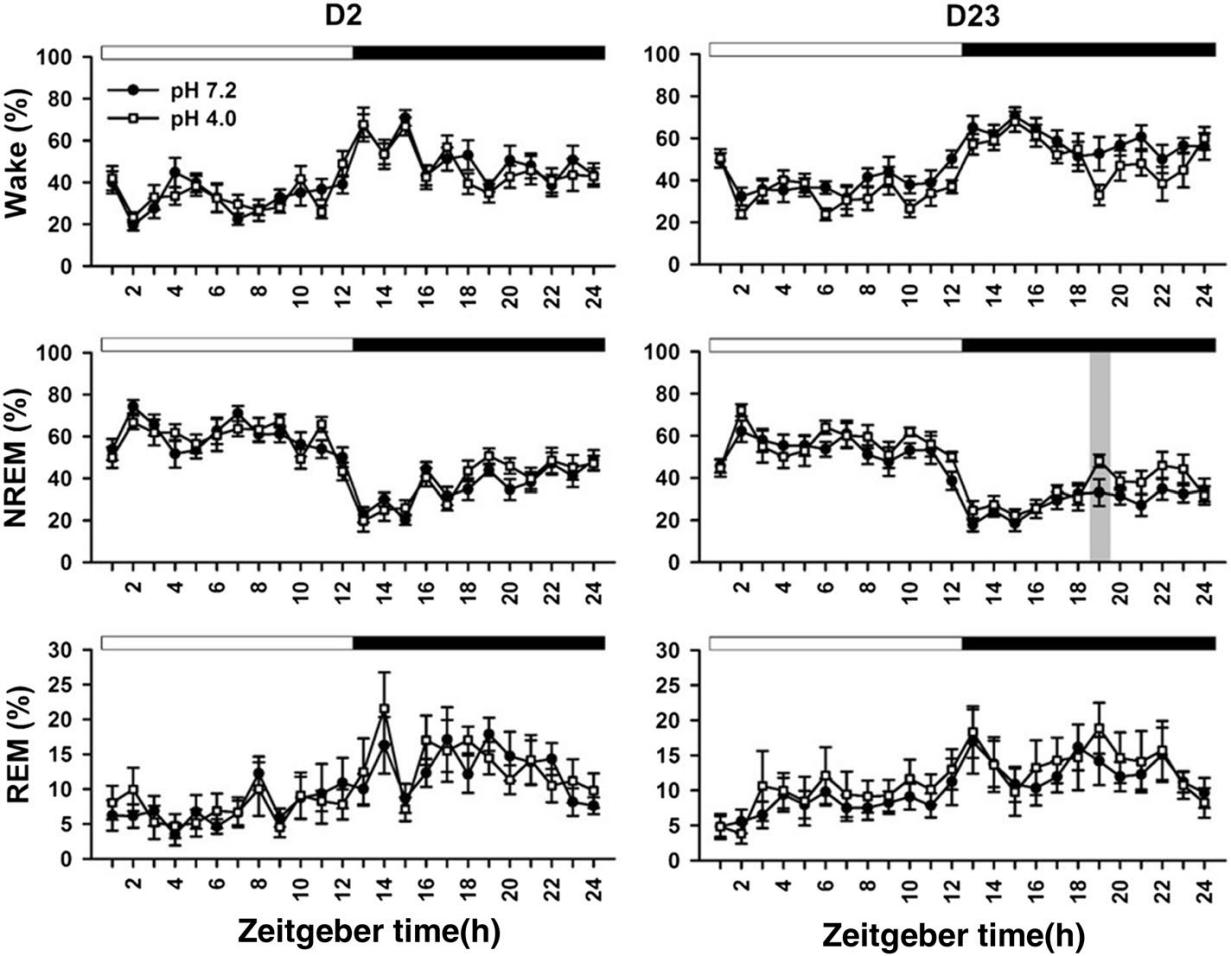
Changes of 3 wake–sleep stages per hour in the light and dark periods at days 2 and 23. Statistical significance between the vehicle group (filled circle) and acid group (open square) is indicated by gray bar. Light and dark periods are indicated with open horizontal bar and filled horizontal bar at the top of each subplot, respectively. Time is modified as zeitgeber time

When we used 3-stage wake–sleep analysis in the two groups at 2 days, there was significant difference in the time factor exclusively (Table 4). There was no significant difference in the factors of treatment or time × treatment at days 2 and 23. At day 23, there was a significant difference in NREM sleep of the dark period between the two groups (Fig. 4).

## Discussion

The present study introduced 10 features with a simple threshold combined with a two-step hierarchical decision tree to characterize wake–sleep stages in rats. In both 5-stage and 3-stage wake–sleep classification, our method presented a high agreement with two experts. In an acid-induced widespread hyperalgesia model, 5-stage wake–sleep classification exhibited subtle sleep disturbance when hyperalgesia developed exclusively. The current study validated our automatic rule-based algorithm on a good performance for wake–sleep classification and effectiveness in the acid-induced hyperalgesia model.

Table 6 summarizes all parameters and performance of this study and previous studies [11, 13, 14, 16, 22] in terms of signals, subject number, epoch length, total epoch number, and proposed methods under the 3-stage analysis. Overall agreements of these methods ranged from 88 to 96%, including 92–99% for wake, 85–97% for NREM, and 79–94% for REM. Among these data, our results demonstrated a high overall agreement (> 94%), and all stages’ agreements exceeded 90% (94.9% for wake, 96.5% for NREM, and 90.48% for REM). Our 3-stage analytic method had optimal performance in terms of high agreement and *κ* value. The present study utilized a minimum number of signals and largest number of subjects (*N* = 20). Amount of epochs used in this study was several to ten folds of previous studies. Thus, the current study strengthens the reliability to validate our automatic scoring method in a simple preparation for sleep recording.

**Table 6:** Comparisons of all available methods for wake–sleep classification

References	Signal	Subjects	Method	Epoch		Agreement (%)			
				Length (s)	Number	Wake	NREM	REM	All
[11]	1 EEG, 1 EMG	5	Logic algorithm	20	19,499	98.7	90.7	93.9	94.3
[13]	1 EEG, 1 EMG	14	Logic algorithm	5	6138	91.8	92.5	79.4	87.9
[14]	2 EEG, 1 EMG	6	Custom SVM	20	10,870	96.5	96.9	95.4	96.8
[16]	1 EEG, 1 EMG	9	Logic algorithm	5	5594	99.3	97.4	90.9	95.9
[17]	2 EEG, 2 EMG, 2 EKG	7	Support SVM	10	20,174	83.45	91.74	61.5	84.39
[22]	1 EEG, 1 EMG, 1 Locomotion	10	Logic algorithm	10	75,801	93.4	85.0	82.5	90.9
Our method	1 EEG, 1 EMG	20	Logic algorithm	10	168,656	94.4	96.5	90.48	94.97

In the scoring method using 1 EEG and 1 EMG, most agreements between experts and automatic scoring from this study and previous studies [11, 16] were higher than 90%. These studies extracted crucial features from EEG and EMG (including alpha band power-related spindle activity and delta power of slow-wave activity) according to raters’ experience. The present study selected features of a previous study [16] to calculate 3 valuable indexes for stages W, N, and R at the first part of the decision tree; afterwards, we further calculated relative power from powers of selected bands as indexes to finely tune threshold at the second part of the decision tree. Agreement of our method (94.97%) was slightly lower than 95.9% of the previous study [16], particularly for the wake stage. This study preserved a comparable agreement using tenfold epochs compared with the previous study. Discrepancies may arise from different recording periods (24-h recording with 12-h light-off period in this study vs. 4-h light-on recording) and epoch amounts (168,656 of this study vs. 5594). Because rats are a nocturnal animal, they usually present quite wake state in the light-on period. The quite wake state is relatively easy to be correctly identified rather than active wake stage in the light-off period. On the other hand, the previous study presents 88.8% agreement from 9327 epochs and 95.9% agreement for 5594 epochs with high confidence between two raters [16]. Highly selected epochs of the previous study may be a reason to explain its high agreement.

Human sleep staging uses epoch of 30 s. However, rats are nocturnal animals with a relative short sleep cycle [16, 20]. Thus, previous studies have selected epoch with a relative short duration for sleep staging in rats, such as 5 [13, 16], 10 [17, 22], or 20 s [11, 14]. In general, a long segment contains valuable wideband information with less sensitivity for transient response. By contrast, a short segment emphasizes on a transient variability exclusively. This study selected an epoch of 10 s as a compromise between valuable information and transient variance [17, 22]. To further extract valuable transient response, the present study designed fine analysis of 5.2-s epochs for each 10-s epoch combined with a rule-based decision tree to determine the behavioral stage [28]. Our results (94.97% agreement) exhibited relatively higher than previous studies (84.39% [17] or 90.9% [22]) in Table 6. Our proposed epoch length and alternative analytic method seem to be beneficial for staging analysis in rats.

**Fig. 5:**
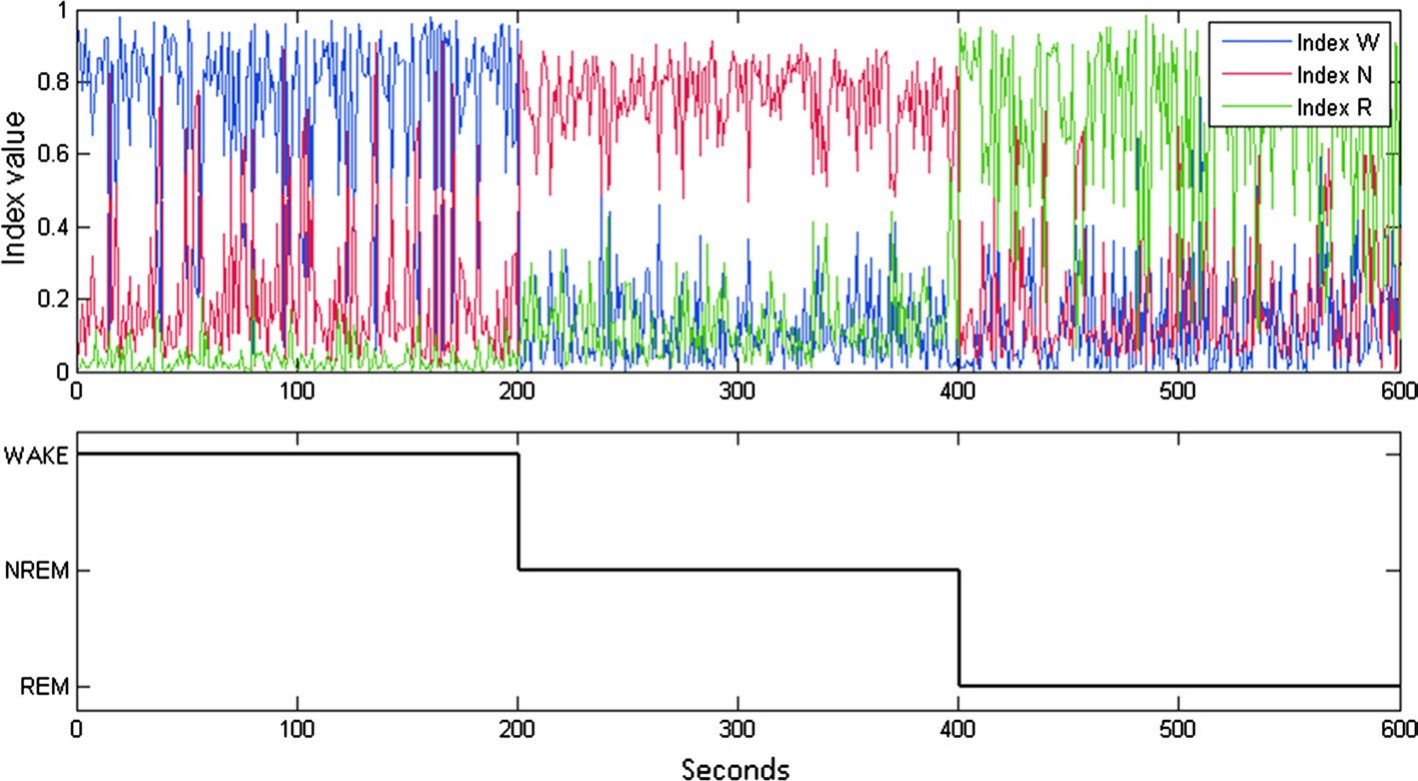
The values of Index_*W*_, Index_*N*_, and Index_*R*_ in the wake, NREM, and REM stages (1st to 200th epochs are wake, 201st to 400th epochs are NREM, and 401st to 600th epochs are REM). Blue line: the value of Index_*W*_; red line: the value of Index_*N*_; green line: the value of Index_*R*_

**Fig. 6:**
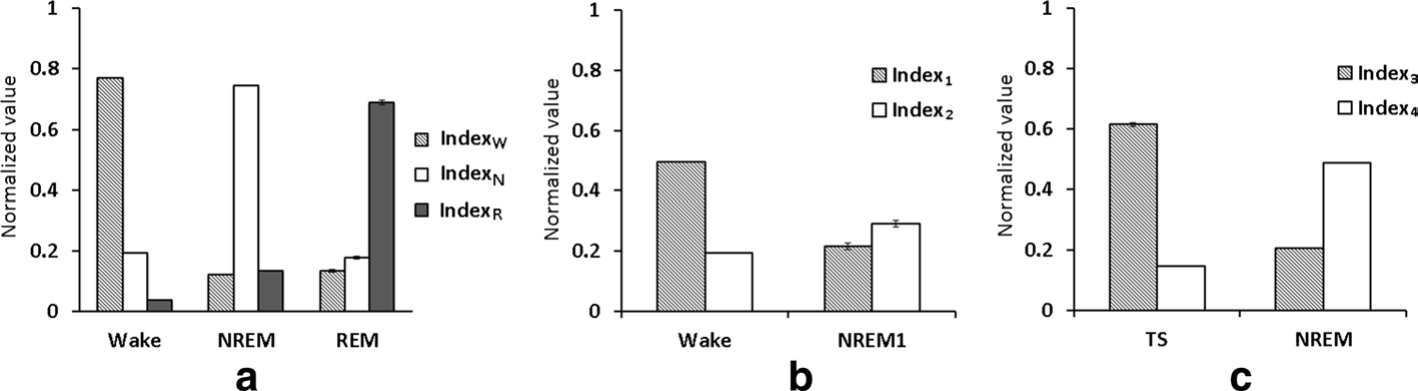
**a** Values of the Index_*W*_, Index_*N*_ and Index_*R*_ in the wake, NREM, and REM stages from the training dataset. **b** Values of the Index_*1*_ and Index_*2*_ in the wake and NREM1 stages from the training dataset. **c** Values of Index_*3*_ and Index_*4*_ from the training dataset

Valuable features play an important role in classification of different behavioral stages. Numerous studies have suggested useful features for staging in EEG, such as band power [29–32], spectral power [29–31], higher-order spectra [33], entropy [30, 34, 35], wavelets coefficient [29, 31, 36, 37], etc. The present study selected certain classic band powers of a rat EEG as features [16, 38], which are common and useful in automatic staging previously [14–16, 18]. We derived several valuable indexes from 10 features through statistical assessment (Figs. 5, 6). Most importantly, normalization of all selected indexes exhibited the advantage of eliminating individual differences [28]. The normalization of all indexes is also helpful for high consistency of the automatic scoring among subjects. The present study exhibited *κ* value of > 0.8 occurring in 95% of rats for the 3-stage scoring and 100% of rats for the 5-stage scoring. These results demonstrated subject-independent robustness of our strategies using band power-related features combined with normalization. In addition to band power of EEG, the wavelet analysis is recently emphasized for non-stationary signal [31, 36]. The contribution of wavelet coefficients on our proposed automated scoring method remains to be determined.

**Fig. 7:**
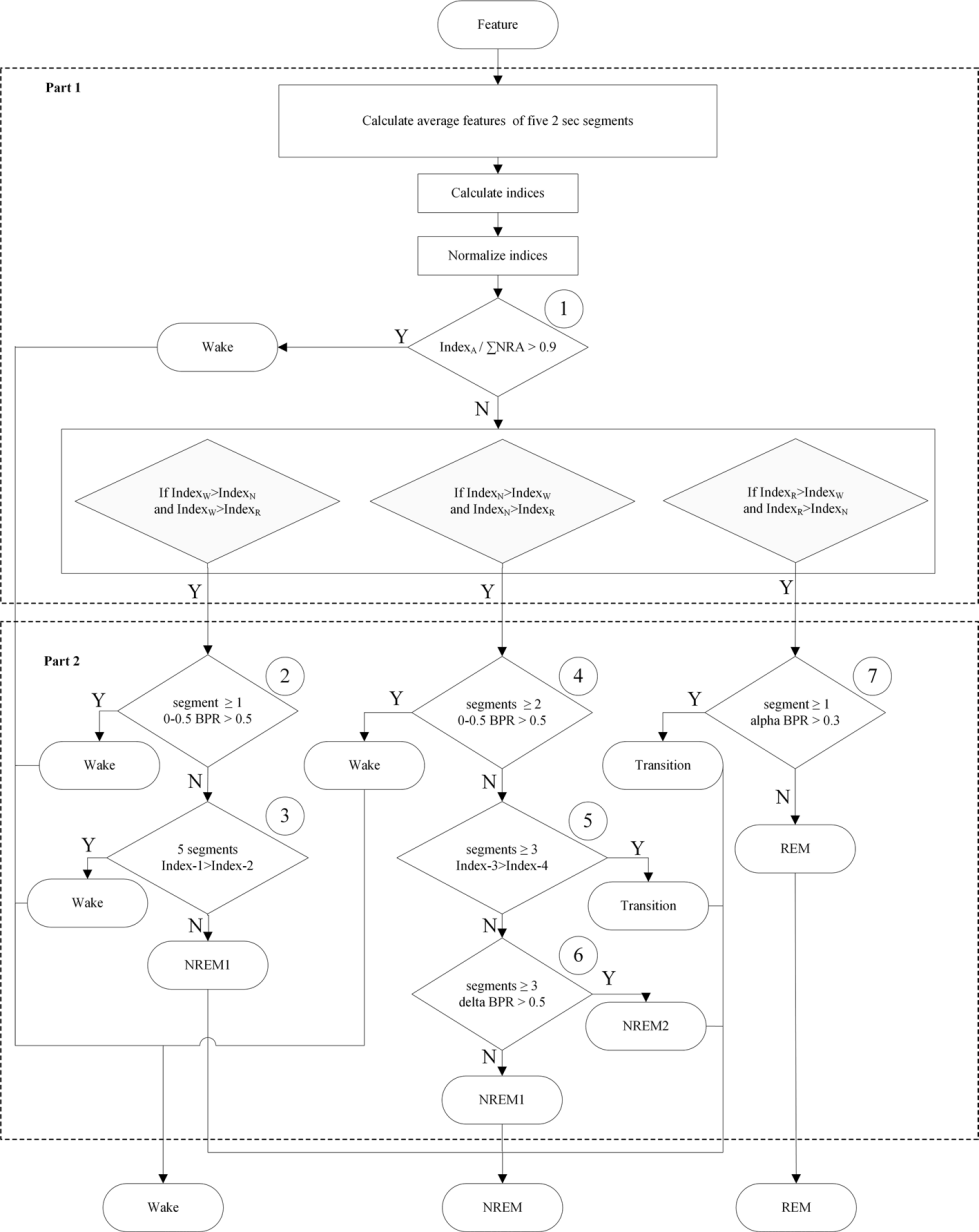
Flow chart of the proposed decision tree. BPR: band power ratio; ∑NRA: Index_*N*_ + Index_*R*_ + Index_*A*_

Previous studies have introduced various classification algorithms, including artificial neural network [29–31, 35–37], decision trees [29, 31], liner discriminant analysis [29, 31, 34], extreme learning machine [32], Gaussian mixture models [33], etc. Accuracies of those classifiers for sleep scoring have a large variance (75–95%). Ideally, a simple classifier is used in the case with excellent representative features. According to statistical evaluation of valuable indexes (Fig. 6), a simple threshold was used in several testing points of the decision tree (Fig. 7) and had a great advantage on reduction of computation power. The present study also modified the decision tree into two principal parts according to valuable features of previous studies [16] and prior experience of experts. Thus, our algorithm was easy to determine two kinds of sleep scorings. Agreements for the 3-stage scoring and 5-stage scoring were 94.97% and 92.32%, respectively. In the present study, *κ* values of almost all subjects were > 0.8 (i.e., excellent agreement) for the two-stage scorings. Based on these results, the present study proposes a new decision tree combined with valuable features for sleep scoring.

The 5-stage wake–sleep classification in rats has been proposed in a previous study with a semiautomatic scoring method [20]. Table 7 shows agreements of the previous study [20] and our method. The present study achieved a better performance in wake (94.4% vs 85.14%), NREM1 (91.13% vs 71.51%), NREM2 (91.26% vs 89.94%), and TS (78.98% vs 72.55%). The performance of REM in this study (90.48%) was slightly less than the previous study (94.52%). Overall agreement (92.32%) of this study was higher than that of the previous method (82.63%). The *κ* value of this study indicated an excellent agreement, and the previous study only exhibited substantial agreement. Taken together, the present study advances automatic scoring technique of the 5-stage analysis.

**Table 7:** Confusion matrix of the agreement by method of Neckelmann et al. (a) and our method (b)

(a)	Automatic staging [20]				
	Wake	NREM1	NREM2	TS	REM
Expert consensus					
Wake	85.14	11.80	1.72	0.02	1.32
NREM1	2.74	71.51	22.90	1.10	1.76
NREM2	0.32	9.12	89.94	0.03	0.59
TS	0.00	17.66	5.49	72.55	4.30
REM	2.94	1.73	0.00	0.81	94.52
Overall agr. (%)	82.63				
Kappa	0.78				

(b)	Automatic staging				
	Wake	NREM1	NREM2	TS	REM
Expert consensus					
Wake	94.4	4.62	0.61	0.18	0.19
NREM1	3.84	91.13	2.65	1.99	0.38
NREM2	1.07	7.31	91.26	0.24	0.11
TS	3.03	14.13	0.10	78.98	3.76
REM	3.45	4.19	0.43	1.46	90.48
Overall agr. (%)	92.32				
Kappa	0.88				

The 3-stage staging method provided little information of slow-wave activity in NREM sleep. Thus, it is difficult to explore interactive change of delta activity and alpha activity during NREM sleep. In a sharp contrast, the 5-stage scoring is able to observe possible alteration between slow-wave activity (delta power) and spindle (alpha power) [20]. At the baseline (day 2), both groups did not differ from each other in terms of PWT and sleep pattern. Rats exhibited decrease NREM2 sleep (slow-wave sleep) followed by increased NREM1 sleep (light sleep) during light period (Fig. 3), which is similar to nocturnal sleep pattern in humans [1]. This cyclic change of NREM1 and NREM2 during light-on period cannot be seen in the 3-stage analysis (Fig. 4). Repetitive acid injection into an unilateral muscle caused widespread hyperalgesia at day 23 [39]. The present study also characterized acid-induced hyperalgesia comorbid with sleep disturbance, i.e., longer NREM1 sleep and shorter NREM2 sleep (Table 4). This phenomenon exists in most humans with chronic widespread pain syndromes, such as fibromyalgia [2]. Taken together, the present study provides additional face validity of acid-induced hyperalgesia model as human’s fibromyalgia and further support on the 5-stage wake–sleep analysis.

## Conclusions

We performed and validated a rule-based automated sleep scoring system in rats. The proposed method exhibits 92.32% agreement in the five-stage scoring and 94.97% agreement in the three-stage scoring with a manually reference from two scorers. Ten features of the EEG and EMG signals were utilized. Normalization of these feature-derived indexes was employed to reduce individual variability. A simple threshold was set to separate different stages. Compared with other classifiers, such as neural networks [12, 24] or linear discriminator analysis [40], the thresholding in this approach is less computationally complex. Our method classified the vast majority of epochs with excellent agreement through high *κ* value. The performance of our proposed five-stage method is superior to existing methods. Because the classification is less computation power and more robustness and consistency, this algorithm can be implanted into a versatile wireless portable monitoring system for real-time analysis in the near future.

## Methods

### Animal preparation and experimental procedure

Adult male Sprague–Dawley rats (*n* = 20, 300–400 g) were used. Rats were raised in a sound-attenuated room with a 12–12 light–dark cycle (06:00–18:00 lights on) and comfortable temperature (25 ± 2 °C). Rats were randomly assigned into a group receiving the vehicle (pH 7.2, *n* = 11) or acid saline (pH 4.0, *n* = 11). The Institutional Animal Care and Use Committee of National Cheng Kung University reviewed and approved the experimental procedures.

The recording electrodes were implanted under pentobarbital anesthesia (60 mg/kg, i.p.). Following anesthesia induction, the rat was placed in a standard stereotaxic apparatus. The dorsal surface of the skull was exposed and cleaned. Seven stainless steel screws were driven bilaterally into the skull overlaying the frontal (2.0 mm anterior to and 2.0 mm lateral to the bregma), parietal (2.0 mm posterior to and 2.0 mm lateral to the bregma), and occipital (6.0 mm posterior to and 2.0 mm lateral to the bregma) regions of the cortex [5]. A reference electrode was implanted 2.0 mm caudal to the lambda. Seven-strand stainless steel microwires (#7935, A-M Systems) were bilaterally inserted into the dorsal neck muscles to record EMG. Monopolar EEG recording and bipolar EMG recording were used. There were two groups: the first group (No. 1–10) had EEG recordings from bilateral frontal lobe and right occipital lobe; the second group (No. 11–20) had EEG recordings from bilateral parietal lobe and right occipital lobe. The occipital EEG is good to pick up hippocampal theta activity for characterizing REM sleep [5]. Following the surgery, the rats were administered antibiotics (chlortetracycline) and housed individually in cages for 1 week of recovery. To allow the rats to become habituated to the experimental apparatus, each animal was placed in the recording environment 1 day prior to the experiment.

Induction of chronic hyperalgesia was described in our previous study [39]. In brief, normal saline (pH 7.2) was adjusted with an 2-(*N*-morpholino)ethanesulfonic acid to pH 4.0 ± 0.1 as acid saline. All rats were briefly anesthetized with vaporized isoflurane (3–5%). The left gastrocnemius muscle was injected with 100-μl neutral saline (vehicle group) or acid saline (acid group) on days 3 and 8.

Hyperalgesia test in terms of paw withdrawal threshold (PWT) has been described in our previous study [39]. Briefly, rats were placed in a Lucite cubicle on an elevated metal grid allowing to stimulate the plantar surface of a paw. Von Frey filaments were applied to the plantar surface of a paw. A “response” to the stimuli was defined as an abrupt lifting of the foot upon application of the von Frey filaments. A trial contained 5 von Frey stimuli. PWT was defined as the lowest force that elicited ≥ 3 withdrawals in 5 consecutive stimuli. PWT of the ipsilateral left hindpaw was measured followed by the contralateral right hindpaw. In the present study, PWTs before the 1st injection (D1) and 14 days after the 2nd injection (D22) were selected to demonstrate effect of repetitive unilateral injection of acid saline eliciting bilateral chronic hyperalgesia. Sleep recording of 26 h (from 5:00 a.m. to 7:00 a.m. of the next day) was performed at day 2 (baseline) and day 23 (severe hyperalgesia) with regard to measures of PWTs [39].

### Sleep recording and stage scoring

Rats were briefly anesthetized with vaporized isoflurane (3–5%). Dental cement was used to fix a recording wire, which contained an amplifier headset, with the connector over the rat’s head. The rat was placed in a transparent acrylic box, and the recording wire was connected into a multichannel commutator (Model#SL-36, Dragonfly Inc., West Virginia, USA) for free movement in the recording box.

A head set contained several N-channel field-effect transistors (MMBF5484, Motorola Semiconductor, USA) to act as a transconductance voltage buffer to reduce possible interference of external electromagnetic field coupling from the recording wire [41]. EEGs of frontal, parietal and occipital cortices were amplified (5000×) and filtered (0.1–70 Hz). EMG was amplified (1000×) in the range of 100–500 Hz. The EEG and EMG were synchronously digitized at different sampling rates (200 and 500 Hz, respectively) through a 12-bit analog–digital converter (PCL-818L, Advantech, Taiwan) connected to an IBM PC-compatible computer. The entire software, including data acquisition and analysis, was developed in MATLAB. The acquired data were stored on a hard disk for subsequent off-line verification.

All sleep recordings from 20 rats were scored visually by two sleep specialists with a 10-s segment (termed the epoch). The training data were randomly selected from two rats (one from the first group and the other one from the second group) and the remaining rats in the two groups were used for testing.

### Feature extraction

The present study used Fast Fourier Transform (FFT) to characterize powers of specific bandwidths. A variety of frequency- and time-domain features were extracted from 2-s non-overlapping segments of the sleep data. Table 8 lists the 10 features used in this study [16, 27].

**Table 8:** Summary of features

Type	Feature	Formula
Spectral power	0–0.5 Hz (EEG)	Mean (power (dB))
	0.5–5 Hz (EEG)	
	6–9 Hz (EEG)	
	10.5–15 Hz (EEG)	
	22–30 Hz (EEG)	
	35–45 HZ (EEG)	
Power ratio	0–0.5 Hz (EEG)	Power (0–0.5)/power (0–30)
	0.5–5 Hz (EEG)	Power (0.5–5)/power (0–30)
	10.5–15 Hz (EEG)	Power (10.5–15)/power (0–30)
EMG energy	Amp M (EMG)	Mean (abs (amp.))

Spectral power (SP): Following FFT, we calculated the mean spectral power (dB) among each frequency band for the EEG (EEG_lo_; 0–0.5 Hz, *δ*; 0.5–5 Hz, *θ*; 6–9 Hz, *α*; 10.5–15 Hz, *β*; 22–30 Hz, *γ*; 35–45 Hz).

1 $${\text{SP}}_{i} = {\text{norm}}\left[ {{\text{SP}}\left( {f_{i} } \right)} \right],\quad {\text{where }}i = 1 - 6$$

Power ratio (PR): Following FFT, we calculated the total spectral power (dB) of 0–30 Hz and the mean power of each frequency band in the EEG. Then, we calculated the ratio of each band power divided by the total power [power (0–30 Hz)] as a feature. Table 8 shows three power ratios as our features (EEG_lo_; 0–0.5 Hz, *δ*; 0.5–5 Hz, *α*; 10.5–15 Hz).

2 $${\text{PR}} = \frac{{{\text{norm}}\left[ {{\text{SP}}\left( {f_{i} } \right)} \right]}}{{{\text{norm}}\left[ {{\text{SP}}\left( {f_{j} } \right)} \right]}},\quad {\text{where }}i = 1 - 3$$

$$0 \le f_{1} < 0.5, \quad 0.5 \le f_{2} < 5, \quad 10.5 \le f_{3} < 15, \quad 0 \le f_{j} \le 30.$$

EMG energy: EMG signal was filtered in the range of 10–100 Hz. The mean value of the absolute amplitude of the filtered EMG in an epoch was calculated from as a feature.

Following feature extraction, normalization of the features was employed to prevent extreme values influencing analysis then to reduce possible individual variability [28]. For each feature, the mean of the maximal 10% data was calculated as the maximum value of the feature, and the mean of the minimal 10% data was calculated as the minimum value of the feature. The procedure for normalization was summarized in the following steps:

- *Step 1* The means of the 10% minimal and maximal values for each feature as the min and max values, respectively, were calculated.
- *Step 2* The min and max values were set as 0 and 1; the other values were then normalized from 0 to 1.
- *Step 3* If the value was higher than 1, the value was specified as 1. If the value was lower than 0, the value was specified as 0.

Two steps are required after the elementary construction of a decision tree: (1) selecting appropriate features for each decision node and (2) setting appropriate threshold of the selected features as the splitting predicates. For the first step, the means and the standard deviations of the analyzed feature corresponding to stages *A* and *B* were ($\bar{A}$, $\bar{B}$) and ($\sigma_{A}$, $\sigma_{B}$), respectively. The distribution distance (DD) of the feature with respect to A and B was calculated through the following equation:

3 $${\text{DD}}\left( {A,B} \right) = \left\{ {\begin{array}{*{20}l} {1 - \frac{{\sigma_{A} + \sigma_{B} }}{{2\left| {\bar{A} - \bar{B}} \right|}}} \\ 0 \\ \end{array} } \right.\begin{array}{*{20}l} {{\text{if}}\, \sigma_{A} + \sigma_{B} \le 2\left| {\overline{A} - \overline{B} } \right|} \\ {\text{else}} \\ \end{array} .$$

A feature with a large DD value indicates a large difference between stages *A* and *B*. Afterwards, a large DD value between features was used to select proper features for each node.

For the second step, the present study set an appropriate value for each feature to clarify stage at each node. The threshold for the feature was obtained by following equation:

$${\text{Threshold}} = \frac{1}{2}\left[ {\left( {{\text{mean }}\left( { 10\% { \hbox{max} }} \right) - \frac{1}{2}{\text{Std }}\left( { 10\% { \hbox{max} }} \right)} \right) + \left( {{\text{mean }}\left( { 10\% { \hbox{min} }} \right) + \frac{1}{2}{\text{Std}}\left( { 10\% { \hbox{min} }} \right)} \right)} \right].$$

### Structure of the decision tree

Figure 7 shows flow chart of the proposed decision tree. The decision tree contained two parts and seven testing points. The first part of the decision tree characterized all 10-s epochs into three conditions, i.e., stages *W*, *N*, and *R*. Afterwards, the second part further classified these epochs into the wake, NREM1, NREM2, TS, and REM stages.

In the first part of the decision tree, we used indexes defined in a previous study to classify an epoch into a condition [16]. The present study determined different ratios of the variables to discriminate each condition as follows:

4 $${\text{Index}}_{W} = \left( {{\text{EMG }} \times \gamma } \right)/\delta ,$$

5 $${\text{Index}}_{N} = \left( {\delta \times \alpha } \right) / \gamma^{2} ,$$

6 $${\text{Index}}_{R} = \theta^{3} /\left( {\delta \times \alpha \times {\text{EMG}}} \right),$$

7 $${\text{Index}}_{A} = {{\left[ {\left( {2 \times {\text{EEG}}_{\text{lo}} } \right) + \beta } \right] \times \gamma } \mathord{\left/ {\vphantom {{\left[ {\left( {2 \times {\text{EEG}}_{\text{lo}} } \right) + \beta } \right] \times \gamma } {\sum {\text{EEG}}}}} \right. \kern-0pt} {\sum {\text{EEG}}}} ,$$

where ∑EEG = *δ* + *θ *+ *α + β + γ.*

A previous study has proposed a short 2-s segment to increase the sensitivity for sleep staging in humans [28]. The current study divided each 10-s epoch into five 2-s segments and then calculated four indexes by the average of five 2-s feature values. The Index_*A*_ was used to detect the artifact stage. The artifacts were characterized by high fluctuation from the signal occasionally accompanied by broadband increases in EEG power [16]. For instance, the artifact was caused by biting or grasping something within a short period in rats. In the first testing point of Fig. 7, the epoch was considered an artifact if the value of the Index_*A*_/∑NRA > 0.9 (where ∑NRA = Index_*N*_ + Index_*R*_ + Index_*A*_). In general, these artifact epochs were considered as wake epochs [22].

Ideally, a good feature set should present great difference in a distinct condition. The Index_*W*_ values would exceed values of the Index_*N*_ and Index_*R*_ in the stage W. The Index_*N*_ values were greater than the values of Index_*W*_ and Index_*R*_ in the stage N, and the Index_*R*_ values should be larger than the values of Index_*W*_ and Index_*N*_ in the stage R. The present study randomly selected 600 10-s epochs from two rats (48-h recording) with staging by two experts as the 3-stage analysis (1st to 200th epochs were wake, 201st to 400th epochs were NREM, and 401st to 600th epochs were REM). A rat contributed 100 epochs for each condition. Three indexes were calculated from normalized values. Figure 5 illustrates the values of Index_*W*_, Index_*N*_, and Index_*R*_ in the wake, NREM, and REM stages, respectively. The index belonging to a particular stage was obviously higher than the other two stages, such as higher Index_*W*_ occurred at the wake stage.

Figure 6a shows values of the Index_*W*_, Index_*N*_ and Index_*R*_ in the wake, NREM, and REM stages from the training dataset. A one-way analysis of variance (ANOVA) [42] was utilized to assess the Index difference under a particular stage, if appropriate, a Bonferroni *t* test [43] was used as a post hoc test. In the wake stage, the Index exhibited significant difference (*F*_2,25875_ = 47807.06, *p *< 0.001). The Index_*W*_ (0.771 ± 0.002) was significantly higher than Index_*N*_ (0.194 ± 0.002) and Index_*R*_ (0.036 ± 0.001). In the NREM stage, the Index exhibited significant difference (*F*_2,26664_ = 78,406.13, *p *< 0.001). The Index_*N*_ (0.744 ± 0.001) was significantly higher than Index_*W*_ (0.122 ± 0.001) and Index_*R*_ (0.134 ± 0.001). In the REM stage, the Index exhibited significant difference (F_2,3600_ = 3229.41, *p *< 0.001). The Index_*R*_ (0.689 ± 0.007) was significantly higher than Index_*W*_ (0.134 ± 0.004) and Index_*N*_ (0.178 ± 0.005).

In the second part of the decision tree, epochs were further divided into the wake, NREM1, NREM2, TS, and REM stages. When a rat exhibited active behavior, extreme movement-induced noise occurred in the EEG signals. In the stage W of the first part, the epoch that low band power ratio (0–0.5 Hz) > 0.5 occurred at ≥ 1.2-s segment was rescored as the wake stage for the 2nd testing point. According to the manual scoring rule, the EEG comprised high frequency, which consisted of predominant theta activity (6–9 Hz) concomitant with a large amplitude EMG in the wake stage; the NREM1 stage presented sleep spindles (*α*; 10.5–15 Hz) and/or median delta wave activity (0.5–5 Hz) less than 50% of the segment accompanied by diminished EMG compared with the wake stage. Therefore, the present study constructed Index_*1*_ and Index_*2*_ as follows:

8 $${\text{Index}}_{1} = {\text{EMG }} \times \gamma / \delta$$

9 $${\text{Index}}_{2} = \alpha \times \delta /\theta$$

Figure 6b shows values of the Index_*1*_ and Index_*2*_ in the wake and NREM1 stages from the training dataset. In the wake stage, the Index_*1*_ (0.496 ± 0.001) was significantly higher than the Index_*2*_ (0.194 ± 0.001; t_70458_ = 179.557, *p *< 0.001). In the NREM1 stage, the Index_*2*_ (0.292 ± 0.011) was significantly higher than the Index_*1*_ (0.216 ± 0.011; t_698_ = -4.969, *p *< 0.001). As shown in the 3rd testing point of the second part decision tree, an epoch that the Index_*1*_ values of all 2-s segments exceed the Index_*2*_ was considered as the wake stage. Otherwise, the epoch was considered as the NREM1 stage.

In the stage N of the first part, the epoch that low band power ratio (0–0.5 Hz) > 0.5 occurred at ≥ 2.2-s segments was rescored as the wake stage for the 4th testing point because an epoch in the stage N probably presented mild delta wave and movement-induced noise simultaneously. Our prior experience expressed ≥ 2 segments with higher low band power ratio as a reasonable index for the wake stage. Subsequently, frontal and parietal EEGs were characterized by a prominent theta rhythm intermittent with short-lasting high-amplitude spindles in the TS. The current study defined Index_*3*_ and Index_*4*_ as follows:

10 $${\text{Index}}_{3} = \theta \times \gamma / \delta$$

11 $${\text{Index}}_{4} { = }\delta / \theta$$

Figure 6c shows values of Index_*3*_ and Index_*4*_ from the training dataset. In the TS, the Index_*3*_ (0.615 ± 0.007) was significantly higher than the Index_*4*_ (0.145 ± 0.000; *t*_3348_ = − 59.757, *p *< 0.001). An epoch that the Index_*3*_ values of ≥ 3.2-s segments exceed the Index_*4*_ was considered as the TS for the 5th testing point. In the NREM1 + NREM2 of the stage N, the Index_*4*_ (0.488 ± 0.001) was significantly higher than the Index_*3*_ (0.206 ± 0.001; *t*_75258_= 171.850, *p *< 0.001). According to prior experience, delta band power of the NREM2 stage was higher than that of the NREM1 stage. The present study considered an epoch as the NREM2 stage if delta band power ratio > 0.5 occurred at ≥ 3.2-s segments for the 6th testing point of the second part decision tree. Otherwise, the epoch was considered as the NREM1 stage.

The TS and REM stages often exhibited theta activity. The TS also embedded higher alpha amplitude of high-amplitude spindle exclusively. In the stage R of the first part decision tree, an epoch that alpha band (10.5–15 Hz) power ratio > 0.3 occurred ≥ 1.2-s segment was considered as the TS. Otherwise, it was considered as the REM stage.

According to the two-part decision tree, the 5-stage scoring was finished. Furthermore, the present study took the NREM1, NREM2 and TS together as the NREM stage for the 3-stage analysis.

### Statistics

**Fig. 8:**
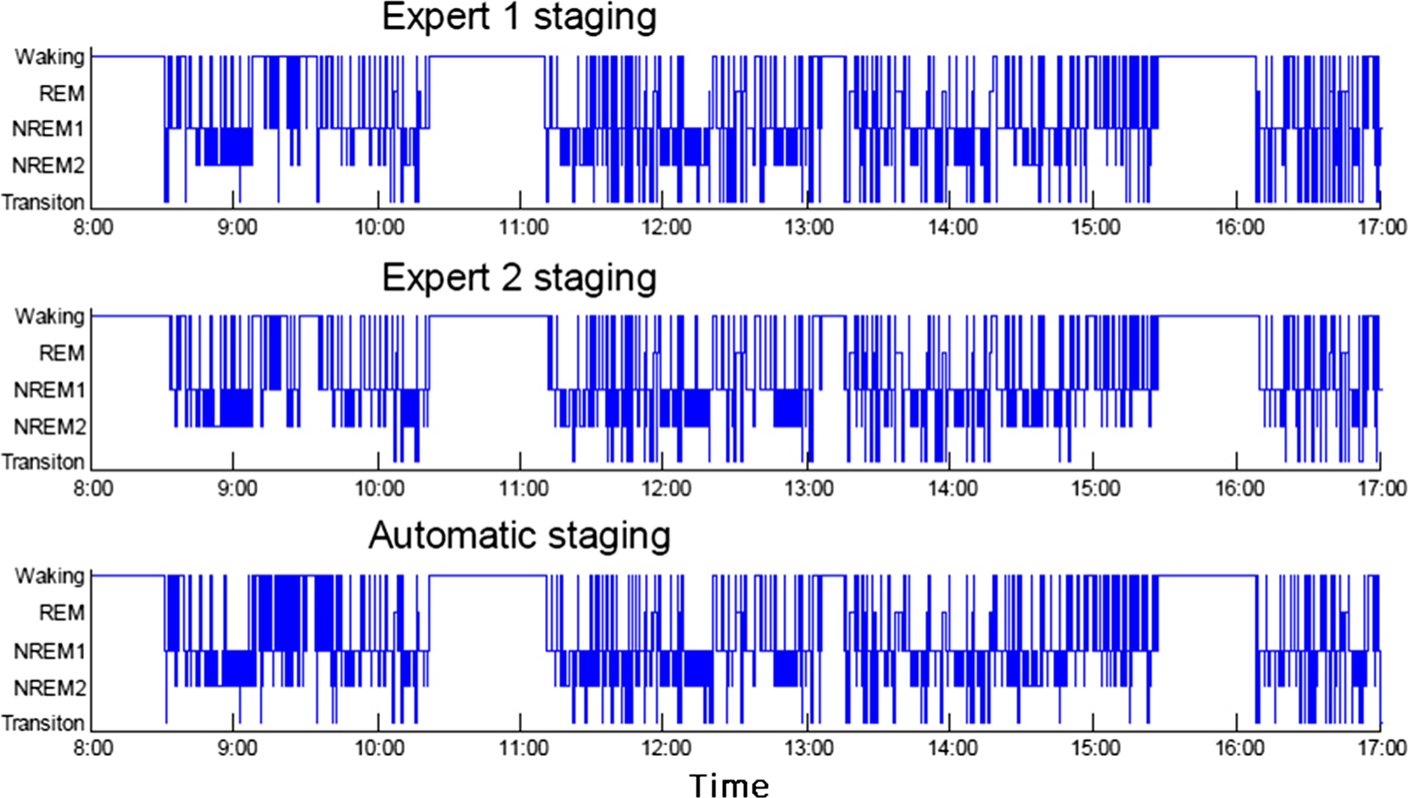
Light phase (8:00 A.M. to 5:00 P.M.) hypnogram of No. 9. Top two panels are manual staging hypnograms from two experts, and bottom panel is a hypnogram from the automatic staging

Two experts used the established rules for visual scoring and did not discuss the data each other. The five-stage (wake, NREM1, NREM2, TS, REM) and three-stage (wake, NREM, REM) scorings were compared here. For the 3-stage analysis, experts considered NREM1, NREM2 and TS as NREM. The automatic staging hypnogram and manual staging were performed. Figure 8 displays three hypnograms scored by expert 1, expert 2 and automatic staging, respectively. The present study compared the automatic scoring with expert 1 and expert 2. For a given epoch, four scoring situations existed: (1) both the two manual scores and the automatic score were identical; (2) the two manual scores were the same but differed from the automatic score; (3) difference in the two manual scorers and the automatic score consenting with a manual scorer; (4) difference among all scorings. The expert consensus scoring defined as epochs in the same sleep stage by the two experts. To reduce possible confusion epochs, epochs with consensus scoring by two experts were used throughout the entire validation procedure.

The performance between the expert consensus and staging method was assessed by numerous indexes, including sensitivity (SE), specificity (SP), number of true positive (PPV), number of true negative (NPV) of each stage, overall agreement, and kappa coefficient (*κ*). Definitions of all indexes were shown below.

12 $${\text{SE }} = \frac{\text{Number of true positives}}{{\left( {{\text{Number of true positives}} + {\text{Number of false negatives}}} \right)}}$$

13 $${\text{SP}} = \frac{\text{Number of true negatives}}{{{\text{Number of true negatives}} + {\text{Number of false positives }}}}$$

14 $${\text{PPV }} = \frac{\text{Number of true positives}}{{\left( {{\text{Number of true positives }} + {\text{Number of false positives}}} \right)}}$$

15 $${\text{NPV}} = \frac{\text{Number of true negatives}}{{\left( {{\text{Number of true negatives }} + {\text{Number of false negatives}}} \right)}}$$

16 $$\kappa = \frac{{{ \Pr }\left( a \right) - { \Pr }\left( e \right)}}{{1 - { \Pr }\left( e \right)}}.$$

Pr(*a*) is the relative observed agreement among scorings, and Pr(*e*) is the hypothetical probability of chance agreement. The Cohen’s kappa coefficient is a statistical measure of the inter-rater agreement [44]. Cohen’s kappa measures the agreement between two scorings who classify *N* items into C mutually exclusive categories. The observed data had been used to calculate the probabilities of each scoring. The interpretation of kappa coefficients by Landis and Koch [45] is as follows: poor agreement with *κ* < 0.00, slight agreement with 0.00 ≤ *κ *≤ 0.20, fair agreement with 0.21 ≤ *κ* ≤ 0.40, moderate agreement with 0.41 ≤ *κ *≤ 0.60, substantial agreement with 0.61 ≤ *κ* ≤ 0.80, and excellent agreement with *κ* > 0.80.

In the acid-induced widespread hyperalgesia, paw withdrawal thresholds of bilateral hindlimbs were analyzed by Friedman repeated measures ANOVA on rank, if appropriate, followed by Dunnett’s test. Changes of 5 wake–sleep stages per hour or 3 wake–sleep stages per hour between the two groups were analyzed using two-way repeated measures ANOVA with one factor repetition, if appropriate, followed by post hoc Bonferroni *t* test. All data of this study were expressed as the means and standard error of the mean (SEM). Level of statistical significance was considered to be *p *< 0.05.

## Data Availability

The datasets generated during and/or analyzed during the current study are available from the corresponding author for reasonable request.

## Abbreviations

EMG: electrotromyogram
EOG: electrooculogram
EEG: electroencephalogram
Wake: wakefulness
NREM: non-rapid eye movement
REM: rapid eye movement
TS: transition sleep
